# Evaluation of a 433 MHz Band Body Sensor Network for Biomedical Applications

**DOI:** 10.3390/s130100898

**Published:** 2013-01-14

**Authors:** Saim Kim, Christian Brendle, Hyun-Young Lee, Marian Walter, Sigrid Gloeggler, Stefan Krueger, Steffen Leonhardt

**Affiliations:** 1 Chair for Medical Information Technology, RWTH Aachen University, Pauwelsstrasse 20, Aachen 52074, Germany; E-Mails: brendle@hia.rwth-aachen.de (C.B.); kriz.lee@ita.rwth-aachen.de (H.-Y.L.); walter@hia.rwth-aachen.de (M.W.); leonhardt@hia.rwth-aachen.de (S.L.); 2 Department of Cardiology, Pneumology, Angiology and Intensive Care, University Hospital Aachen, Pauwelsstrasse 30, Aachen 52074, Germany; E-Mails: sgloeggler@ukaachen.de (S.G.); stkrueger@ukaachen.de (S.K.)

**Keywords:** Body Sensor Network (BSN), 433 MHz ISM band, wireless transmission, sensors, performance evaluation, communication, healthcare, packet loss rate

## Abstract

Body sensor networks (BSN) are an important research topic due to various advantages over conventional measurement equipment. One main advantage is the feasibility to deploy a BSN system for 24/7 health monitoring applications. The requirements for such an application are miniaturization of the network nodes and the use of wireless data transmission technologies to ensure wearability and ease of use. Therefore, the reliability of such a system depends on the quality of the wireless data transmission. At present, most BSNs use ZigBee or other IEEE 802.15.4 based transmission technologies. Here, we evaluated the performance of a wireless transmission system of a novel BSN for biomedical applications in the 433 MHz ISM band, called Integrated Posture and Activity NEtwork by Medit Aachen (IPANEMA) BSN. The 433 MHz ISM band is used mostly by implanted sensors and thus allows easy integration of such into the BSN. Multiple measurement scenarios have been assessed, including varying antenna orientations, transmission distances and the number of network participants. The mean packet loss rate (PLR) was 0.63% for a single slave, which is comparable to IEEE 802.15.4 BSNs in the proximity of Bluetooth or WiFi networks. Secondly, an enhanced version is evaluated during on-body measurements with five slaves. The mean PLR results show a comparable good performance for measurements on a treadmill (2.5%), an outdoor track (3.4%) and in a climate chamber (1.5%).

## 1. Introduction

Body Sensor Networks (BSNs) have been investigated for more than a decade [1] and an increasing number of groups are exploring new applications for BSNs [2,3]. From their initial application in military scenarios [4], BSNs are now a focus in biomedical engineering [1,5–8].

The impact of demographic changes and increasing costs of medical care place a large economic burden on healthcare systems worldwide. Due to their versatility and mobility, BSNs offer new solutions for personal healthcare, without loss of quality of treatment [9]. Applications that previously were not possible due to complicated, costly and bulky equipment are now moving from a stationary hospital-type environment to a more personal environment [9–12]. As described by Kjeldskov *et al.*, the healthcare domain can benefit from context awareness for ubiquitous computing [13]. Therefore, a body sensor is an enabling technology to sense the context of a user.

The monitoring of chronic diseases is an important area of research, including cardiovascular monitoring, diabetes management and rehabilitation of Parkinson's patients [8,14-17]. Small, unobtrusive and battery-driven sensor nodes are used to measure various vital parameters; some are even integrated into textiles. Nevertheless, to enable routine use of BSNs, some remaining problems need to be addressed, e.g., the use of wireless communication technologies [18]. These facilitate communication between network nodes and external devices (such as PDAs or PCs) and also significantly reduce the amount of cabling required. Although radio frequency (RF) transmission is the most commonly used technology, use of the human body as a transmission medium is also feasible [19]. Nevertheless, mobile wireless communication is prone to external influence factors, such as a change in channel conditions caused by movement or location change, and proximity to the human body [20–22].

In contrast to most BSNs, we decided not to use ZigBee as a wireless communication interface because of the interference due to the overlap in frequency band with WiFi and Bluetooth in Europe [23,24]. The only available ZigBee channel outside of the 2.4 GHz range in Europe is at 868 MHz and has a data rate of 20 kbps, which is not sufficient for telemedical monitoring applications. Instead, we use a 433 MHz band transceiver (CC1101, Texas Instruments Inc., Dallas, TX, USA), which is also less prone to electromagnetic shadowing effects by the body itself [21]. Reduced data rates compared with 2.4 GHz transmission systems, e.g., WiFi or Bluetooth, are not critical for biomedical applications [2]. Of all existing BSN systems, only a few operate outside the 2.4 GHz range [25,26]. Currently, the most prominent use of the sub-1 GHz frequencies for biomedical applications is by medical implants in the Medical Implant Communication Service (MICS) band in 402–405 MHz, such as pacemakers. As miniaturization continues and energy efficiency improves, the integration of implantable sensors will be the next logical step in the development of medical body sensor networks [27]. Therefore, our use of this transmission band will facilitate the future integration of implant sensors such as glucose sensors for diabetes, pressure sensor for hydrocephalus and cardiac monitoring systems [28–30].

In this paper, we present the evaluation of novel wireless data transmission of a BSN in the 433 MHz ISM band. At the first, the physical transmission quality and the data transmission quality of the second generation of IPANEMA nodes is examined as a baseline. A signal analysis was performed with a spectrum analyzer, and the packet loss rate (PLR) was estimated taking into account variation in distance and antenna orientation. The results lead to the improved IPANEMA node generation 2.5, which included major hardware and network management changes. Finally, the transmission performance is assessed with four on-body sensors and one ambient sensor with twelve participants while running on a treadmill, running on an outdoor track and sitting in a climate chamber.

## 2. Methods and Material

### 2.1. System Architecture

The aim of the IPANEMA BSN is to provide a wearable and flexible platform to enable mobile measurements in a wide range of medical and health-oriented application scenarios. So far, two applications have been explored: a cardiac monitoring system and a hydration status monitoring system [31,32]. The modular hardware and software concept facilitates adaptation and extension with new sensors and actuators. The design of the IPANEMA wireless sensor node generation 2 is based on the previous MEDIT BSN [31,32]. The focus of the redesign was a significant reduction in size (−33%) and weight (−69%). The use of lithium polymer battery technology instead of nickel metal hybrid batteries had a significant impact. This allowed the use of a smaller housing and thus improved the user comfort during measurements. The main functional units are:

- Microcontroller (MSP430F1611, Texas Instruments Inc., Dallas, TX, USA)
- Power management (TPS61131, Texas Instruments Inc., Dallas, TX, USA)
- Wireless Transceiver (CC1101, Texas Instruments Inc., Dallas, TX, USA)
- Extension port (Microstac12, Erni Electronics GmbH, Adelberg, Germany)

**Figure 1.**
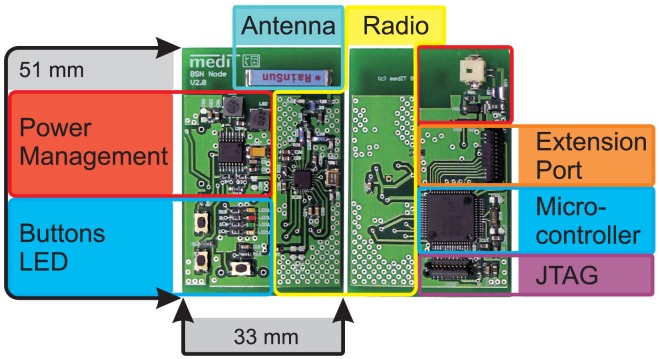
Functional units on the circuit board of an IPANEMA node generation 2.

**Figure 2.**
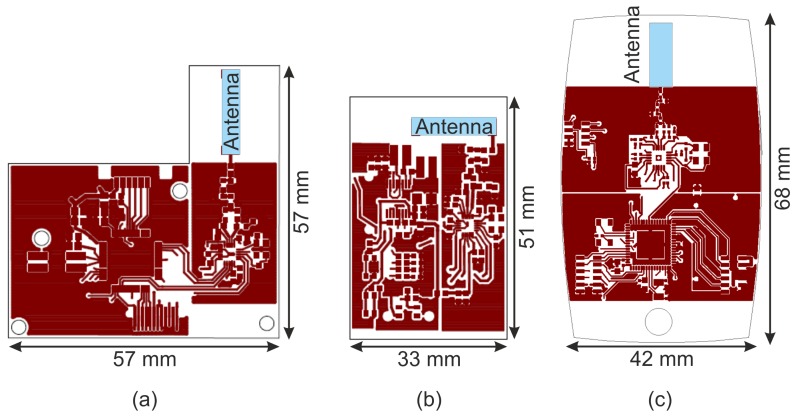
Drawing showing the difference in size between (**a**) the MedIT BSN; (**b**) the IPANEMA BSN nodes generation 2 and (**c**) generation 2.5. Both IPANEMA 2 and 2.5 fit into the same housing.

**Figure 3.**
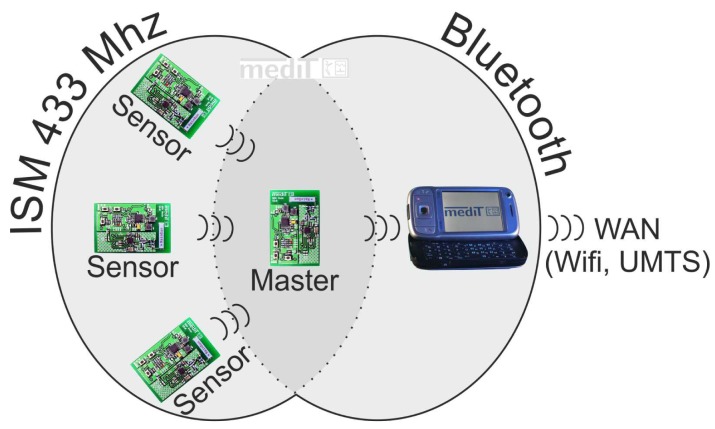
IPANEMA star-shaped network with three sensor slaves and one master module. Data are relayed to a PDA that can send the data over a WiFi or UMTS connection to a back-end server.

Figure 1 shows the arrangement of the functional units on the circuit board of an IPANEMA node generation 2. The components have sleep modes to increase the energy efficiency of the system and thus the run time. As mentioned before, the radio interface of the IPANEMA nodes is based on the highly flexible sub 2 GHz transceiver CC1101. It was configured to work within the European ISM band at 433 MHz. Furthermore, the hardware is compatible to MICS band transceivers, which facilitates the integration of medical implants in future revisions. The channel spacing was set to 200 kHz with a data rate of 250 kbps with a minimum-shift-keying (MSK) modulation and 0 dBm output power. Similar BSN systems in the 433 MHz ISM band (Mica2 and BTnode) offered a significantly lower transmission rate of only 38.4 kbps and have been discontinued [26,33]. Adjacent channels were unused to accommodate the increased channel bandwidth due to the higher bit rate with respect to the channel spacing. All measurements were performed with a single system on a single channel. The settings were derived using the Smart RF studio software (Texas Instruments Inc.,Dallas, TX, USA). A multilayer chip antenna (AN1603-433, Rainsun Enterprise Co., Ltd., Taipei, Taiwan) was used on-board to further reduce the size of the IPANEMA node. The location and orientation of the antenna on the base node is shown in Figure 2. During preliminary test measurements, we noted a change in the transmission reliability, possibly due to a side-effect of size reduction. The IPANEMA network is star-shaped with one network master and a variable number of slave sensor nodes (depending on the sensor type and transmission frequency). The same hardware base platform is used for both types of network nodes as shown in Figure 3. A similar approach was used by Ying *et al.* for an IEEE 802.15.4 based BSN [17]. However, their approach uses mainly local data storage and local analysis of data. Our master node has a Bluetooth module instead of a sensor. This facilitates communication with off-the-shelf equipments, e.g., PCs and smart phones. From there, data may be sent to a server for in-depth analysis and/or to a medical professional. In addition, the master node sends out regular time beacons to synchronize the local clocks of the slave nodes to the master clock, similar to [34]. This is accomplished by calculating the offset between the master clock and the local clock. The local clock uses a 32-bit counter driven by a 32 kHz crystal for a stable time base with millisecond resolution. Data packets from slave nodes are received over the 433 MHz interface and relayed to the Bluetooth interface. Data are framed with a sequence number to detect missing data packets. An identifier in the data packet is used to identify the unique slave address and sensor type of each network node. On the software side, a “round-robin software architecture” is used for the program flow control: a set of flags is checked in an infinite main loop [35]. These flags are set to signalize different events, such as analog-digital-conversion finished, and time synchronization beacon received. All hardware-related interfaces, for example SPI communication, are abstracted in the form of a hardware abstraction layer. This facilitates code reuse on previous and future hardware generations.

### 2.2. Improvements in IPANEMA Generation 2.5

A new generation of IPANEMA nodes was developed to improve the transmission performance of the previous generation 2. Changes included both hardware and software components.

#### 2.2.1. Hardware

Major changes included the RF components and the power management. The matching network was optimized as suggested by the manufacturer and the chip antenna was replaced (CAN4311129200431K, YAGEO corp., Taiwan). Also, the layout was optimized to reduce directivity issues of the previous generation by placing the antenna perpendicular to the circuit board edge as shown in Figure 2. Additionally, a smaller battery was chosen to prevent shadowing due to partial covering of the antenna. Other changes to the power management included a new IC (LTC3558, Linear Technology corp.,Milpitas, CA, USA), which enabled in-housing charging through a micro USB connector.

#### 2.2.2. Network

**Figure 4.**
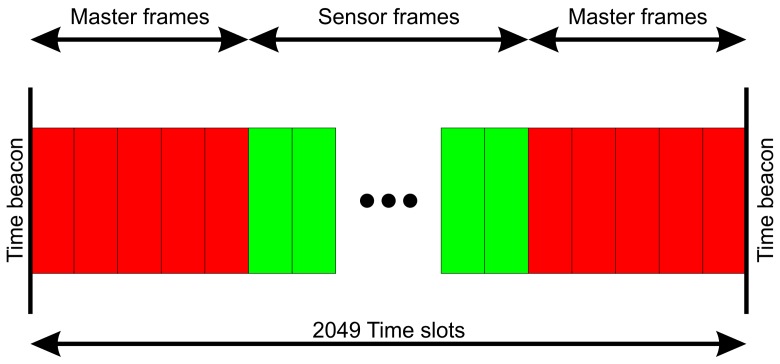
TDMA scheme of the IPANEMA generation 2.5 nodes.

Transmission performance measurements with more than one IPANEMA generation 2 sensor suggested improvements of the network management layer of the firmware. A time division multiple access (TDMA) scheme was implemented based on the existing time synchronization mechanism. A so-called superframe is defined by adjacent time synchronization beacons of the master node as shown in Figure 4. The superframe is divided into 2,049 time slots of 2.4 ms length. This allows to transmit a maximum length packet of 61 bytes plus a safety period before and after, to accommodate clock jitter. Each sensor has exclusive time slots assigned to it. The number is defined by the type of sensor and its sampling frequency. Furthermore, 5 time slots before and after a time synchronization beacon are reserved for the master node for command transmission.

### 2.3. Validation of Wireless Communication Interface

Even though the transmission distances are relatively short in BSNs, the wearer of such a system might continuously change his location, as well as the relative position of the nodes to each other. Also, the nodes might be on both sides of the wearer, whereby the body obscures radio wave propagation. Thus, the channel properties are constantly changing [21].

Also, the human body in close proximity to the node partly absorbs the transmission energy and thus restricts the maximum transmission energy [36]. Therefore, it is important to establish a reliable communication channel between the sensor nodes and the master node. For the analysis, both first and second-generation nodes have been used: a first-generation module was used as a network master. The second-generation module was used as the system under test (SUT), as a slave node. The validation was performed in a standard laboratory environment as a network testbed to assess the real-world properties in a defined scenario.

#### 2.3.1. Spectrum Analysis

First, a spectrum analysis was performed to estimate the raw signal quality in terms of center frequency and spectrum shape. A spectrum analyzer measures the frequency dependent transmission power over a wide bandwidth. In general, the transmission power (*L_P_*) is defined as Equation (1) and the signal-to-noise ratio (*SNR*) is defined as Equation (2).

(1) $$L_{P}=10\;\text{log}\frac{P}{P_{0}}[\text{dBm}]$$

(2) $$\text{SNR}=\frac{L_{\text{Signal}}}{N_{0}}[dB]$$

*P*Measured transmission power*P*_0_Reference power of 1 mW*L_Signal_*Signal power*N*_0_Noise power

A pseudo-random noise generator (PNG) is implemented on the IPANEMA node to simulate random payload for the spectrum analysis. Of the several possibilities to generate random numbers on a microcontroller [37], we chose a linear feedback shift register that does not require any additional hardware. A primitive 16th order generator polynome *G*(*x*) with a 16-bit unsigned integer variable as shown in Equation (3) was chosen.

(3) $$G(X)=X^{16}+X^{12}+X^{3}+X+1$$

A simulated maximum payload of 40 bytes was then generated and sent via the radio interface. A wide band antenna (HyperLOG 3080, Aronia AG,Strickscheid, Germany) connected to the spectrum analyzer (RSA6114A, Tektronix, Inc.,Beaverton, OR, USA) was used as a measurement antenna. Both antennas were aligned parallel to each other, with 2 cm between each antenna. The spectrum shape and time variant spectrum changes were evaluated to validate the RF performance.

#### 2.3.2. PLR Measurements

The second approach to validate the wireless communication interface was the determination of the standard channel properties, such as the PLR. The PLR is often used as an indicator for the current channel conditions [24,38]. The PLR is defined by:

(4) $$\text{PLR}=\frac{\#\text{of erroneous packets}}{\#\text{of total packets}}.100\%$$

A PLR of up to 15% is considered as tolerable for BSNs according to [39].

Because the firmware of the master node was unchanged for the PLR measurements, the data flow was not altered. Instead, data were tapped on the hardware level by listening to the Universal Asynchronous Receiver/Transmitter (UART) interface between the microcontroller and the Bluetooth add-on module.

**Figure 5.**
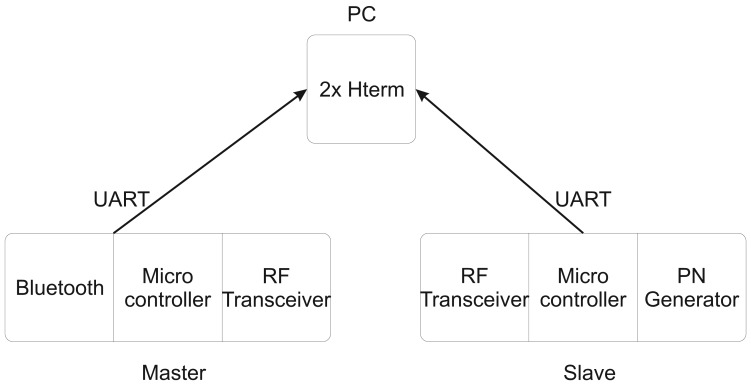
Diagram of the data interception setup of the master and slave node. A terminal program called *Hterm* is used to record the serial data stream.

This was not possible for the slave node, since the PNG had to be integrated into the firmware. Instead, every generated PN series was sent to the regular data handling routines and then to the RF transceiver, as well as over the second wired UART interface of the microcontroller to the PC (Figure 5). The advantage was that the generated PN series was readily available as a semicolon separated byte stream on the PC. On the other hand, data from the master were only available in the regular data packet format including the protocol overhead, such as time stamp, packet counter, node identifier, *etc*. Extracting the PN series in the payload was a multi-step process. To facilitate the payload extraction, a special byte frame was added by the slave firmware to the PN series. Both data streams were logged and saved on a PC for later offline analysis with a program implemented in MATLAB (R2007b, The MathWorks Inc.,Natick, MA, USA).

The transceivers were configured to use no forward-error-correction, interleaving or data whitening. Also, no error correcting code was used in the communication protocol to obtain the raw bit stream. The PLR measurement series included the following measurements:

- Single/dual slave node lab setup with variation of distance and orientation
- Five slave nodes on-body measurement

Analysis of the measurement data can be divided into two consecutive steps:

1. Identification of packet loss: Have all packets been received by the master node?
2. Identification of bit errors: Is the received PN series identical to the transmitted series?

The result of the analysis is a matrix consisting of:

- Error type
- Packet size
- Position of the bit error in the payload
- Original PN series

Bit errors were detected by comparing the received packets from the master node with the original PN series sent by the slave node during the lab setup measurements. Packet loss was detected by checking the sequence number of the packets. Every false or missing packet increased the number of erroneous packets used in the calculation of the PLR as shown in Equation (4). The mean (*μ*) and the standard deviation (*σ*) of the PLR were calculated according to Equations (5) and (6) with *x* as the PLR of a single measurement and *n* as the total number of measurements.

(5) $$\mu =\frac{\overset{n}{\underset{i=1}{\text{∑}}}x_{i}}{n}$$

(6) $$\sigma =\sqrt{\frac{\overset{n}{\underset{i=1}{\text{∑}}}{\left(x_{i}-\mu \right)}^{2}}{n-1}}$$

##### Lab setup Measurements

**Figure 6.**
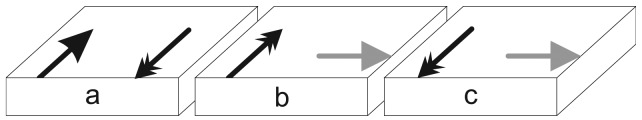
Antenna orientation for measurement of the packet loss rate of IPANEMA generation 2 nodes. The double-headed arrows represent the master antenna and the single-headed arrows represent the slave antenna. Black arrows represent antennas on the top of the circuit board, and grey arrows the antennas on the bottom of the circuit board.

First, a measurement series with one master and one slave module at multiple distances was conducted. The distance was varied in 0.5 m steps from 0.5 m up to 2 m, which is the expected transmission range for a BSN with sensors worn on-body or implanted. Additionally, the antenna orientations were varied according to Figure 6 in three different alignments (a, b, c). The measurement duration was 10 min for each antenna orientation with a transfer of at least 492 data packets (average payload transmission rate of 32.55 bytes/s and 47.83 bytes/s including the protocol overhead) (Table 1). Furthermore, 7 PLR measurements were performed with two slave nodes active at the same time. Transmission ranges were 1 and 1.5 m between the slave nodes and the master, with the same three orientations as described above.

##### On-Body Measurements

The on-body measurements were approved by the local ethic committee (DIMDI 00016588). The measurements were performed in three different settings:

1. Indoor treadmill
2. Outdoor running track
3. Climate chamber

**Figure 7.**
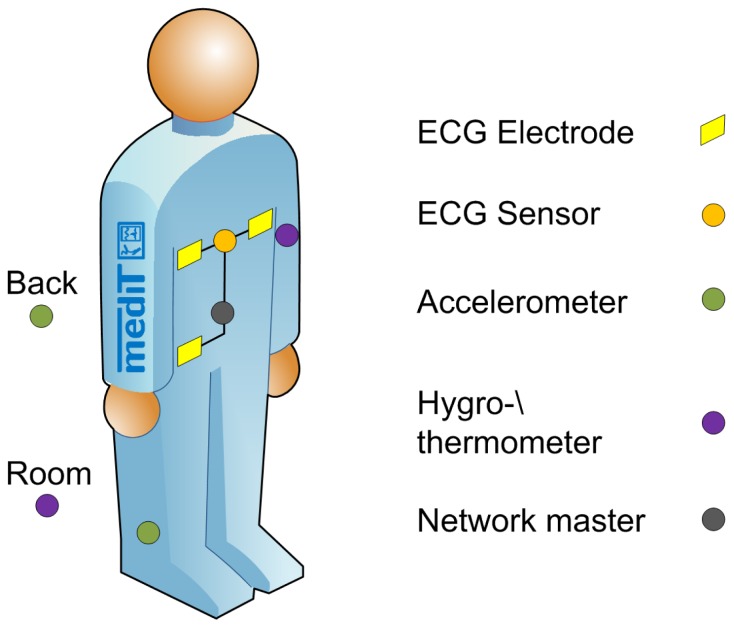
Position of the IPANEMA generation 2.5 sensor nodes on the body.

Each participant was equipped with five sensors, located as shown in Figure 7. Details of each sensor can be found in Table 2. The master node was placed on a belt above the umbilicus. The running speed was 9.6 (± 2) km/h indoors and 10.8 (± 1) km/h outdoors. During the climate chamber measurement the participant were sitting calmly. Table 3 shows the biometric data of the participants.

## 3. Results and Discussion

### 3.1. Spectrum Analysis

**Figure 8.**
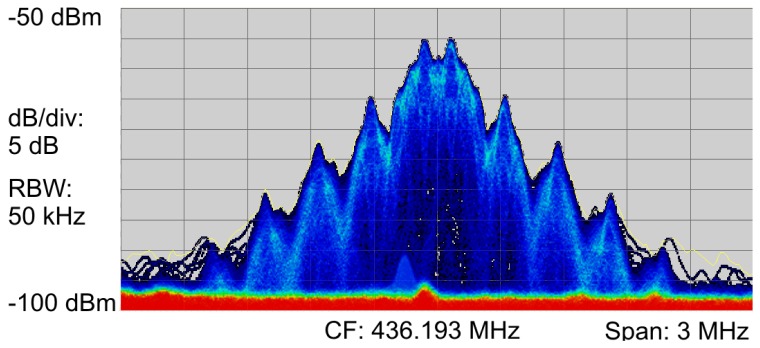
Measured power spectrum of the MSK modulated signal with a PN-generated random payload and a resolution bandwidth (RBW) of 50 kHz.

**Figure 9.**
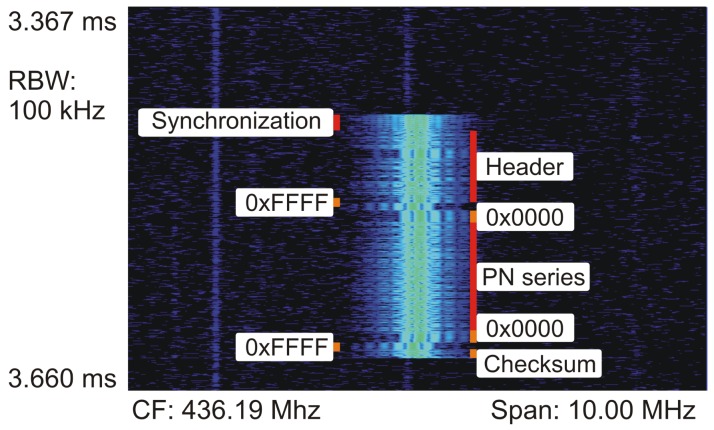
Measured spectrogram of the transmission of one packet. First, the sync word was sent to signalize the transmission of a new packet to the receiver and to ensure that the channel was clear. This was done by switching between 0 and 1. Then, the protocol header was sent, including information such as packet size and recipient address. The actual payload was marked with four flag bytes (0×0000 0×FFFF, and vice versa) before and after, with a visible spectrum shift. The transmission was concluded with the checksum.

Figure 8 shows the time averaged spectrum from the signal analyzer. The shape of the spectrum was as expected for an MSK modulated signal [40]. The double peaks were a result of the modulation technique, representing the bit value of “0” and “1”. Since Figure 8 shows a time-averaged spectrum, both “0” and “1” can be seen in the graph [41]. There were no visible side peaks or variations in shape that might have caused problems on the receiver side. The SNR was about 40 dB and the bandwidth about 3 MHz. Several spectrograms were recorded to verify that there are no time-dependent changes in spectrum shape due to external interferences. Figure 9 is an example of such a measurement. The time axis runs from top to bottom and covers a period of 0.293 ms. The actual transmission duration of the packet was about 0.185 ms. The packet components are clearly visible (see Figure 9). The center frequency (CF) was as expected at 436.19 MHz for channel number *X* = 16 with a channel spacing of *Y* = 199.951172 kHz.

(7) CF=433MHz+X×YkHz

(8) $$CF_{16}=433\;\text{MHz}+16\times 199.951172\;\text{kHz}$$

(9) $$CF_{16}=436.19\;\text{MHz}$$

We wanted to assess the performance under the expected normal measurement conditions in a laboratory. Thus, we did not perform the measurements in an anechoic chamber. All effects that might have a negative effect on the wireless data transmission, e.g., influence of reflections, external radio sources, interference from human activity in the lab and/or attenuation caused by other materials in the lab, such as measurement equipment and other electronic devices (e.g., computers), are included in the measurement results. Therefore, the results represent the performance that can be expected under real-life conditions.

### 3.2. PLR Measurements

#### 3.2.1. Lab Setup Measurements

**Figure 10.**
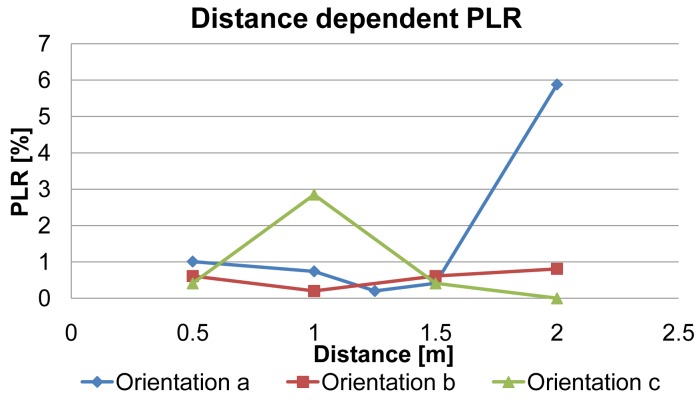
Packet loss rate (PLR) measurements using the three antenna alignments as described in Figure 6.

**Figure 11.**
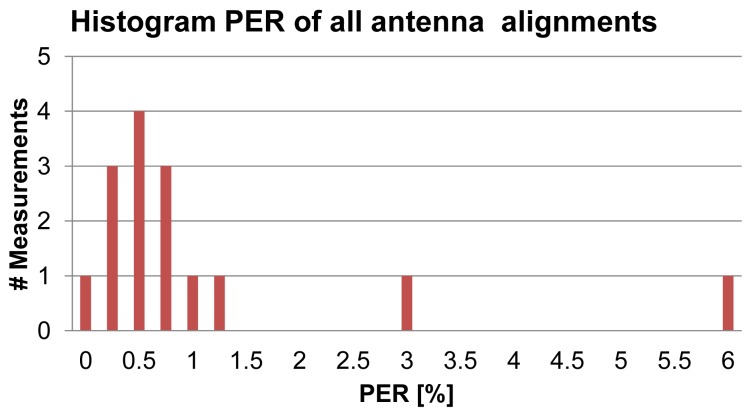
Histogram of packet loss rate (PLR) during the 15 × 10-min measurements of all three antenna alignments of IPANEMA generation 2.

**Figure 12.**
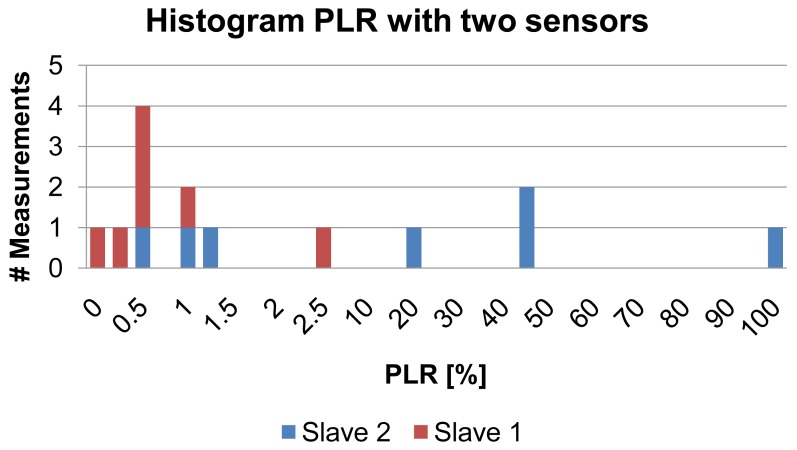
Histogram of packet loss rate (PLR) measurements using two sensor IPANEMA generation 2 nodes.

The results of the PLR measurements for all three antenna alignments with IPANEMA generation 2 nodes are shown in Figure 10. There is no clear increase in the PLR for distances measured between 0.5 m and 2 m for all antenna orientations of the SUT, except for the two outliers for orientation **a** at 2 m and orientation **c** at 1 m. Most likely these outliers have been caused by the directional characteristic of the chip antenna. Thus, Table 4 shows the influence of the antenna orientation on the PLR. Orientation **a** and **c** show a much higher mean value and a much higher standard deviation. As stated before, packet errors can be caused by two distinct failure modes: complete packet loss and bit errors. In our measurements, only complete packet losses occurred. There were no single or multiple bit errors in the received data packets. Every single packet was successfully created on the slave node and transferred to the RF transceiver, as the data were logged over a secondary SPI interface from the microcontroller. Therefore, every received packet was complete and error free. Figure 11 is a histogram of the PLR measurement series. The distribution can be divided into two groups: the first group had a PLR in the range of 0%–1.25% with a mean of 0.63% and a standard deviation of 0.34%. This PLR is in a range where a retransmission request, based on a missing transmission sequence number, would be feasible. Thus, the reliability would be greatly improved since 87% of the PLR measurements were within this range. Also, the PLR is comparable to the PLR of a ZigBee system in the proximity of Bluetooth (2–3 m) and WiFi (over 9 m) according to [38]. Nevertheless, the two outliers had a PLR of 3% and 6%, respectively, implying that retransmission would not be efficient in those cases. The PLR measurement with two sensor nodes yielded a slightly different result (Figure 12). Although the PLR of one sensor was comparable to the results of the first measurement series, the other was much higher (Table 5). During measurement, the second sensor had extended periods in which no packet was successfully transmitted. Nevertheless, the microcontroller was continuously generating PN numbers as they were received over the tapped wire interface, showing that the program was still running correctly. Packet collision cannot be an explanation either, even though the transmission rate was the same for both sensors, since a collision handling with random delay was used. Therefore, both sensor nodes would have been equally affected. An improved collision avoidance technique or time division multiple access (TDMA) scheme may effectively reduce the PLR in the multiple sensor scenario and increase energy efficiency [42].

#### 3.2.2. On-body Measurements

The on-body measurements were performed with the revised IPANEMA generation 2.5 as described in Section 2.2. As expected the PLR has improved significantly during all measurements as shown in Table 6. The sensors with a constant orientation relative to the master node (ECG, ACC2BACK) have the lowest PLR for each measurement. The highest PLR was measured from the accelerometer that was placed on the left ankle with a mean of 3.51%. Indoor PLR is slightly lower compared with the outdoor results while the climate chamber measurement shows the lowest PLR for all sensors. This might be due to the change of the measurement environment: the treadmill was located in a temporary lab space in a container. Instead of concrete, the walls were made of steel and therefore increasing the reflection of the radio waves compared with the outdoor measurement, thus improving the PLR. Furthermore, the climate room measurement was also performed in an isolated metal chamber. Additionally, the participants did not move during the measurement.

### 3.3. Comparison with ZigBee-based Systems

There are many ZigBee-related publications that assess the radio performance of sensor networks under laboratory conditions and under the influence of co-interference sources, e.g., WiFi/Bluetooth [23,24,43–45]. On the other hand, there are numerous publications that describe the application of a ZigBee-based BSN for sports monitoring [46–48]. However, the authors of this work have not found any publication about the assessment of the radio performance of a ZigBee BSN during sports. Therefore, we are comparing the performance of our system to the existing literature data that are based on laboratory measurements. We are assuming that the PLR under laboratory conditions will always be superior to real-life conditions in the field. Thus, these measurements will be treated as the lower bound of the PLR of ZigBee BSNs, because negative effects due to movement of the sensor nodes and shadowing of the body are not included.

Xia *et al.* have evaluated the distance related PLR of a ZigBee sensor network (MICA2, Crossbow Inc.,Milpitas, CA, USA). In the range of up to two meters they have measured a PLR in 4%–10% at the identical output power of 0 dBm [43]. Calvacanti *et al.* showed through simulations that WiFi traffic can increase the PER to 52%–63% for medical BSNs. Sikora *et al.* used a 802.15.4 radio transceiver (CC2420, Texas Instruments Inc.,Dallas, TX, USA). Bluetooth transmissions resulted in a PLR of 10%. Furthermore, WiFi traffic caused a PLR of 35%–95% depending on the channel overlapping [24]. Similar results have been shown by Petrova *et al.* with a PLR in 10%–80% [44]. Shopov *et al.* measured a PLR in 4%–37% depending on the type of WiFi traffic (video transmission and secure file transfer) [45].

In comparison, the PLR of our system ranged in 0.52%–4.18% throughout all measurements. The usage of the 433 MHz ISM band preempted WiFi or Bluetooth interference. The change of measurement conditions in terms of location (indoor *vs*. outdoor) and/or activity (dynamic *vs*. static) did not significantly influence the PLR. Thus, the reliability of the radio transmission of the IPANEMA BSN as a sports monitoring system was demonstrated under real-life conditions.

## 4. Conclusions

We have evaluated the wireless transmission system of a novel BSN working in the 433 MHz ISM band. Multiple test scenarios were evaluated to analyze different factors that might influence channel quality, e.g., antenna orientation, number of slave nodes, and on-body environment. There was no indication that the transmission range of up to 2 m had a significant influence on the PLR in the transmission distances tested.

The only failure mode discovered during the measurements was the loss of packets. No single or multiple bit errors occurred during the lab setup measurements. The PLR was mainly in the range of up to 1.25%, making it feasible to implement software-based retransmission routines to improve transmission reliability. Also, the PLR is comparable to the PLR of ZigBee systems in the proximity of Bluetooth and WiFi systems, which nowadays are found in both laboratory and home environments [38] and below the 15% limit as proposed in [39].

The hardware and network management changes for the IPANEMA generation 2.5 resulted in a significant reduction of the PLR rate showing the success of the improvements despite the increase of network nodes from two to five, and an increased sampling rate of the sensors. The IPANEMA generation 2.5 was deployed for over 70 h in three different measurement settings. Therefore, the feasibility of operating a BSN in the 433 MHz band was proven.

The lessons we have learned during the development of the IPANEMA BSN were: the design of a wireless sensor node requires careful placement of components on the circuit board (antenna, matching network, external components, e.g., battery, *etc*.) with respect to finished setup including the housing. Also, the antenna should be radiating homogenously to allow free placement around the body. Furthermore, the network management has to be able to handle multiple sensor data streams. We have shown that a TDMA scheme is one possible solution.

Future work will be towards the integration of implants into the IPANEMA BSN allowing to extend and to create new application scenarios.

## Figures and Tables

**Table 1.** Average number of packets sent and average transmission rate including protocol overhead for all lab setup packet loss rate measurements with IPANEMA generation 2 nodes. n = the total number of generated packets for each setup.

**Orientation**	**Avg. Packets Send**	**Avg. TX Rate [B/s]**	**n**
a	502.6	47.81	2513
b	493.25	47.87	1973

**Table 2.** Sensor location and sampling frequencies of the on-nody measurements with IPANEMA generation 2.5 nodes. One packet may contain more than one sample (data plus time stamp). The ambient hygro-/thermometer was placed in proximity of the runner, not on the body.

**Sensor**	**Location**	**Sampl. Freq. [Hz]**	**Packet Length [bytes]**
ECG	Chest	512	54
Accelerometer	Left ankle lateral	50	42
Accelerometer	Back above coccyx	50	42
Hygro-/thermometer	Left upper arm lateral	0.5	46
Hygro-/thermometer	Room	0.5	46

**Table 3.** Biometric data of the twelve participants.

**Parameter**	**Mean Value**	**± Standard Deviation**
Age (a)	29	± 4
Height (cm)	181	± 5
Weight (kg)	73	± 7

**Table 4.** Statistical analysis of the antenna orientation dependency of the packet loss rate. n = the total number of generated packets.

**Antenna Orientation**	*μ* **PLR [%]**	*σ* **PLR [%]**	**n**
a	1.65	2.39	2513
b	0.56	0.26	1973
c	0.92	1.30	1969

**Table 5.** Data from measurement of PLR measurement with two IPANEMA generation 2 nodes. n = the total number of generated packets.

**Sensor Number**	*μ* **PLR [%]**	*σ* **PLR [%]**	**n**
1	0.75	0.83	3454
2	30.39	36.48	3454

**Table 6.** PLR rates for the on-body measurements with IPANEMA generation 2.5 nodes. Please note that the ambient hygro-/thermometer was not used during the outdoor measurements. The number of indoor transmitted packets is larger because more indoor measurements were performed than climate chamber and outdoor measurements.

**Sensor**		**Indoor** **PLR [%]**	**Outdoor** **PLR [%]**	**Climate** **PLR [%]**
ECG	Mean	2.10	3.61	0.52
	Std.	7.48	7.25	1.66
	n	2,155,776	396,735	1,677,680
ACC1FOOT	Mean	3.61	4.18	2.75
	Std.	6.63	6.06	3.98
	n	471,164	82,589	346,555
ACC2BACK	Mean	2.04	2.07	1.10
	Std.	5.49	4.42	1.65
	n	469,639	67,776	345,739
HYGRO1SKIN	Mean	2.71	3.71	1.68
	Std.	5.84	8.67	1.90
	n	19,207	1366	15,617
HYGRO2ROOM	Mean	2.11	**-**	1.45
	Std.	4.92	**-**	1.71
	n	21,977	**-**	16,099
